# Supplementary material for: Two High-Quality Cygnus Genome Assemblies Reveal Genomic Variations Associated with Plumage Color
Source: Int J Mol Sci. 2023 Nov 29;24(23):16953. doi: 10.3390/ijms242316953 (PMC10707585; doi:10.3390/ijms242316953)
Supplement: Supplementary file 1 [file ijms-24-16953-s001.zip › Supplementary Figures and Tables.pdf]

Supporting Information for

Two high-quality *Cygnus* genome assemblies reveal genomic variations associated with plumage color

Yuqing Chong [1,†], Xiaolong Tu [2,*,†], Ying Lu [1], Zhendong Gao [1], Xiaoming He [1], Jieyun Hong [1], Jiao Wu [1], DongDong Wu [2], Dongmei Xi [1], Weidong Deng [1,*]

This file includes:

Figures S1 to S5

Tables S1 to S19

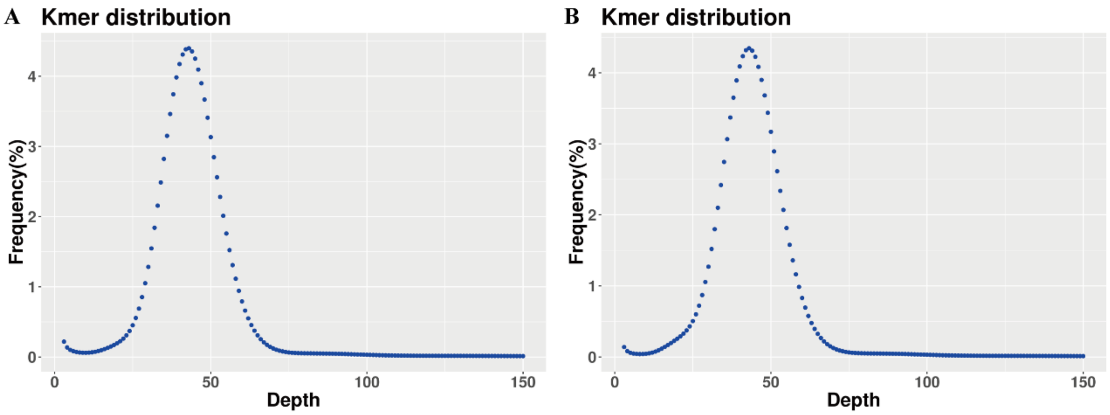

Figure S1. The K-mer distribution diagram for the *C. olor* (A), and the *C. atratus* (B).

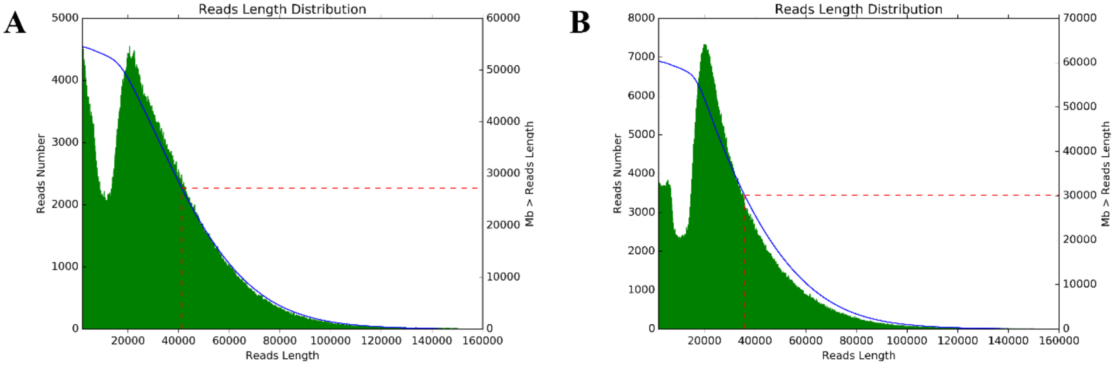

Figure S2. The distribution of Nanopore clean reads length. A, The distribution of clean reads length of the *C. olor*; B, The distribution of clean reads length of the *C. atratus*.

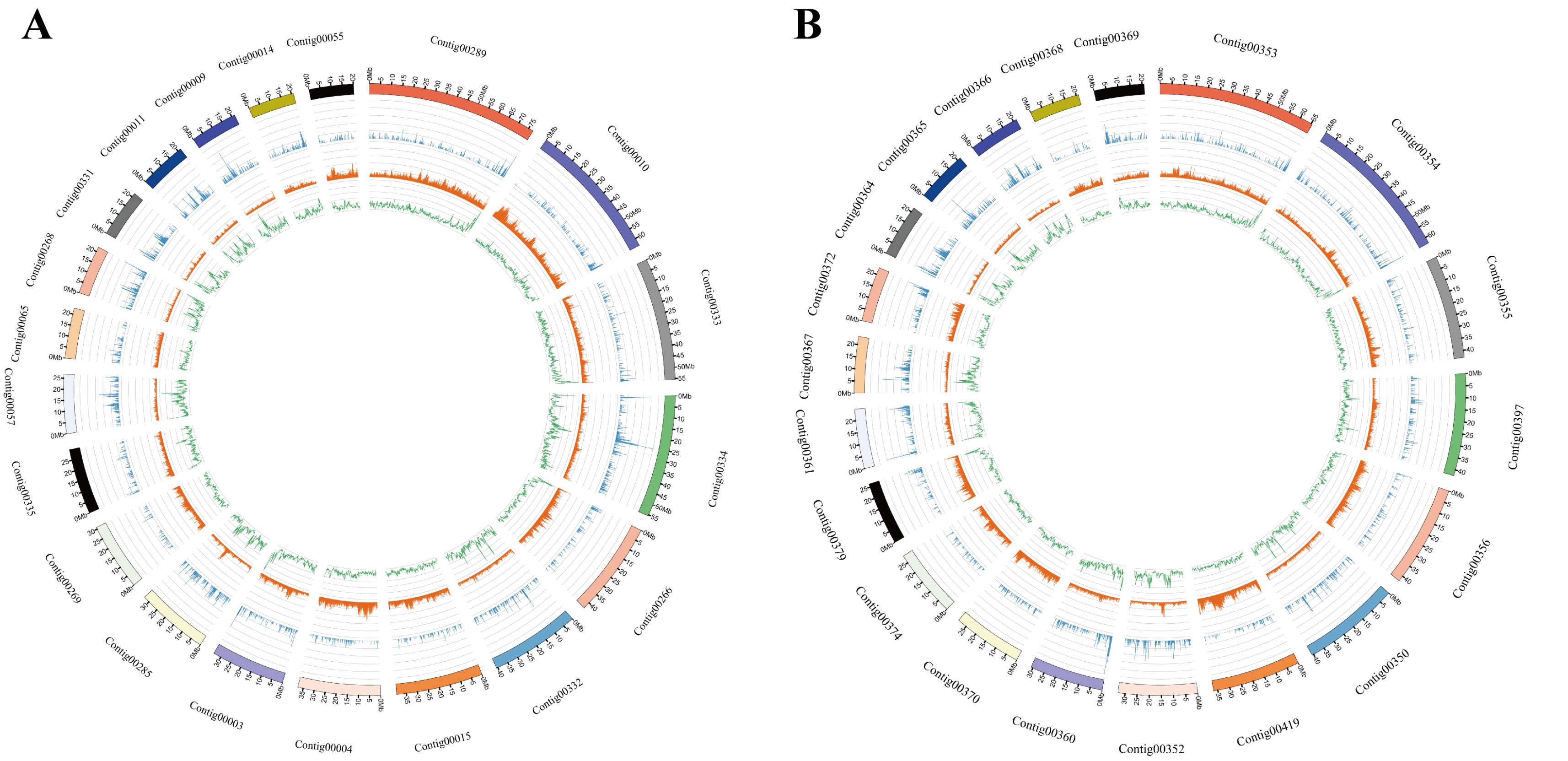

Figure S3. Genome of the *C. olor* and *C. atratus*. A, Circos plot of *C. olor* genomic features. B, Circos plot of *C. atratus* genomic features. Outer to inner: pseudochromosome, gene density (100-kb window) (with higher gene density indicated by higher blue column), repeat sequence (100-kb window), and GC content (100-kb window).

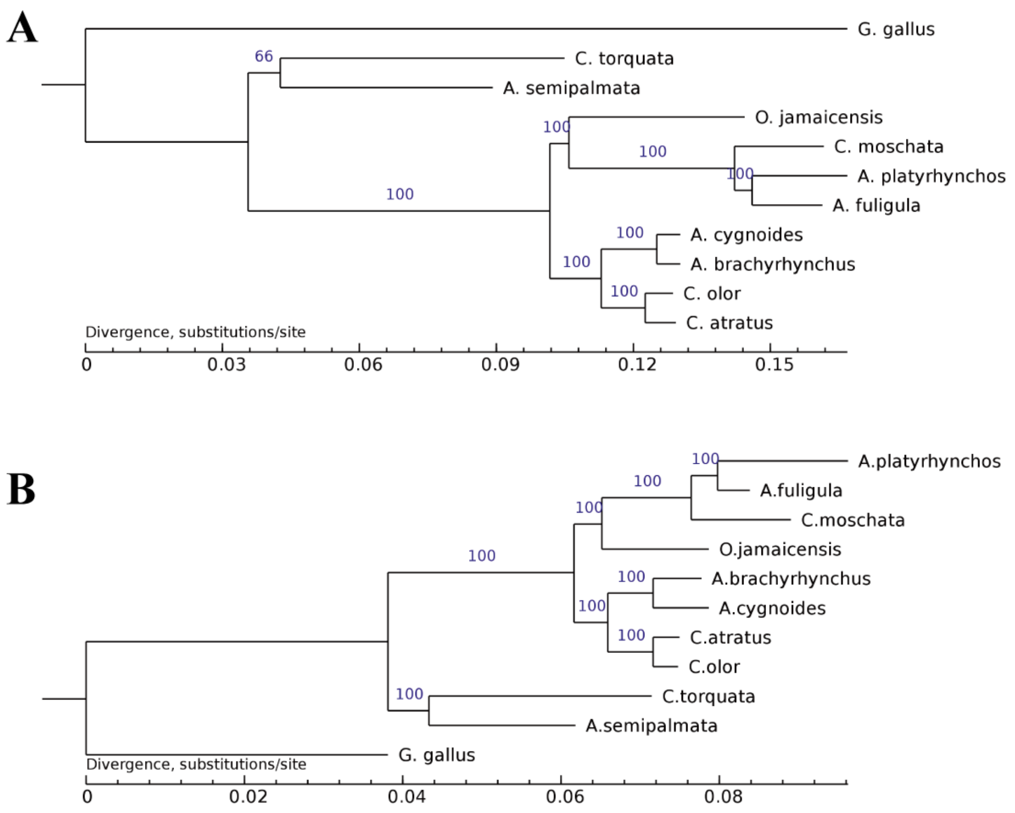

Figure S4. Phylogenetic analysis of *C. olor* and *C. atratus* with other species. A, phylogenetic tree constructed based on 4-fold degenerate sites. B, phylogenetic tree constructed based on CDS sequence.

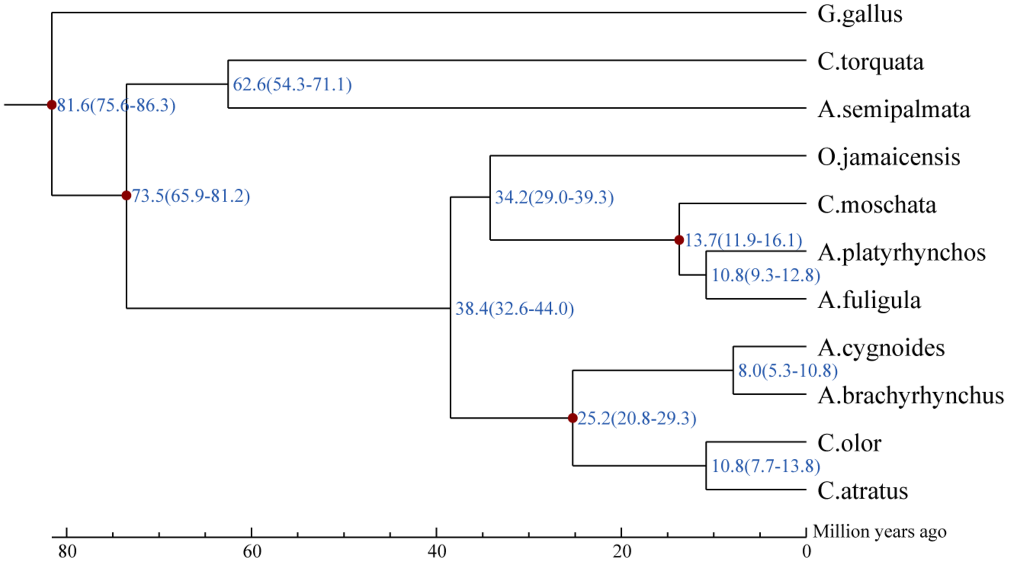

Figure S5. Divergence time analysis of *C. olor* and *C. atratus* with other species.

Table S1. The result of Illumina Hiseq reads.

Species	Library (bp)	Data (Gb)	GC (%)	Q20 (%)	Q30 (%)	Depth (×)
C. olor	350	65.56	46.15	95.61	90.7	53.38
C. atratus	350	62.75	43.18	96.68	92.21	51.5

Table S2. Statistics of C. olor and C. atratus genome survey.

**Parameter**	***C. olor***	***C.atratu***
K-mer	21	21
K-mer number	56,748,688,331	54,326,325,329
K-mer depth	42.01	42.1
Filtered *K-mer* number	51,600,734,780	51,292,910,399
Genome size	1.23	1.22
Heterozygous ratio	0.11%	0.14%
Repeat	14.76%	14.09%

Table S3. Statistical results of Nanopore sequencing data of C. olor and C. atratus.

Species	Data type	SeqNum	SumBase (bp)	N50Len (bp)	N90Len (bp)	MeanLen (bp)	MaxLen (bp)	MeanQual
C. olor	Raw data	2,777,117	63,201,308,418	40,606	18,397	22,757	331,957	7.56
	Clean data	1,734,438	54,688,747,783	41,316	19,305	31,531	320,873	9
C. atratus	Raw data	3,027,341	65,583,872,446	35,405	17,606	21,663	478,493	8.06
	Clean data	2,071,429	60,390,205,766	35,925	18,275	29,153	355,349	9.5

SeqNum, the number of sequences; SumBase, the total number of bases; N50Len: the length of data N50; N90Len: the length of data N90; MeanLen: the average reads length; MaxLen: the longest reads length; MeanQual: the average reads quality value.

Table S4. The distribution of Nanopore data length of *C. olor* and *C. atratus.*

Species	Length	ReadsNum	TotalLength	Percent	AveLength
C. olor	2,000~5,000	119,192	408,995,957	0.74%	3,431
	5,000~10,000	136,615	990,450,853	1.81%	7,250
	10,000~20,000	294,559	4,646,497,038	8.49%	15,774
	20,000~30,000	395,104	9,785,237,130	17.89%	24,766
	30,000~40,000	294,467	10,225,647,395	18.69%	34,726
	40,000~50,000	200,167	8,933,448,596	16.33%	44,630
	50,000~60,000	126,369	6,899,679,915	12.61%	54,599
	60,000~70,000	76,442	4,933,733,219	9.02%	64,542
	70,000~80,000	43,062	3,208,621,186	5.86%	74,512
	>=80,000	48,461	4,656,436,494	8.51%	96,086
C. atratus	2000~5000	109,360	382,147,045	0.63%	3,494
	5000~10000	148,912	1,075,951,322	1.78%	7,225
	10000~20000	425,647	6,900,973,403	11.42%	16,213
	20000~30000	591,311	14,516,394,951	24.03%	24,550
	30000~40000	339,431	11,727,619,211	19.41%	34,551
	40000~50000	199,849	8,906,030,654	14.74%	44,564
	50000~60000	117,446	6,407,374,286	10.60%	54,556
	60000~70000	66,703	4,303,655,168	7.12%	64,520
	70000~80000	36,243	2,698,858,366	4.46%	74,466
	>=80000	36,527	3,471,201,360	5.74%	95,031

Table S5. Statistical information of second-generation sequencing data alignment.

Species	Total reads	Mapped reads	Mapped (%)	Properly mapped reads	Properly mapped (%)
C. olor	437,801,026	435,447,876	99.46	406,886,910	92.94
C. atratus	418,912,758	416,300,170	99.38	405,847,144	96.88

Total reads, the number of clean reads; Mapped reads, the number of clean reads located to the reference genome; Mapped (%), the percentage of clean reads mapped to the reference genome in all clean reads; Properly mapped reads, the number of clean reads that both pair reads mapped to the reference genome.

Table S6. Evaluation results of the core gene integrity.

Species	Number of 458 CEG* present in assembly	% of 458 CEGs present in assemblies	Number of 248 highly conserved CEGs present	% of 248 highly conserved CEGs present
C. olor	450	98.25%	243	97.98%
C. atratus	447	97.60%	239	96.37%

Number of 458 CEG* present in assembly, the number of 458 genes found in the assembled genome in the CEGMA v2.5 database; % of 458 CEGs present in assemblies, the proportion of conserved genes contained in the assembled genome in 458 conserved genes; Number of 248 highly conserved CEGs present, the number of 248 highly conserved genes found in the genome; % of 248 highly conserved CEGs present, the highly conserved genes contained in the assembled genome account for the proportion of 248 highly conserved genes

Table S7. Evaluation results of BUSCO.

Species	Complete BUSCOs	Complete and single-copy BUSCOs	Complete and duplicated BUSCOs	Fragmented BUSCOs	Missing BUSCOs	Total Lineage BUSCOs
C. olor	2546 (98.45%)	2534 (97.99%)	12 (0.46%)	20 (0.77%)	20 (0.77%)	2,586
C. atratus	2542 (98.30%)	2530 (97.83%)	12 (0.46%)	22 (0.85%)	22 (0.85%)	2,586

Complete BUSCOs, the number of complete genes found; Complete and single copy BUSCOs, the number of single copy genes; Complete and duplicated BUSCOs, the number of multi copy genes; Fragmented BUSCOs, the number of incomplete prediction genes; Missing BUSCOs, the number of unpredicted genes.

Table S8. Repeat sequence prediction for *C. olor* and *C. atratus.*

Species	Type	Number	Length	Rate (%)
C. olor	ClassI	279,541	81,820,333	7.28
	ClassI/DIRS	905	60,863	0.01
	ClassI/LARD	33,988	9,051,253	0.81
	ClassI/LINE	190,987	58,339,998	5.19
	ClassI/LTR/Copia	438	70,803	0.01
	ClassI/LTR/Gypsy	6,398	553,594	0.05
	ClassI/LTR/Unknown	41,246	18,247,630	1.62
	ClassI/PLE	3,377	1,345,259	0.12
	ClassI/SINE	1,574	215,330	0.02
	ClassI/TRIM	152	87,732	0.01
	ClassI/Unknown	476	73,870	0.01
	ClassII	41,729	3,268,808	0.29
	ClassII/Crypton	688	40,913	0
	ClassII/Helitron	2,263	199,931	0.02
	ClassII/MITE	10	1,667	0
	ClassII/Maverick	2,408	355,119	0.03
	ClassII/TIR	22,899	2,030,997	0.18
	ClassII/Unknown	13,461	954,340	0.08
	PotentialHostGene	485	144,549	0.01
	SSR	2,375	1,439,824	0.13
	Unknown	5,686	1,243,017	0.11
	Total	329,816	86,692,218	7.71
C. atratus	ClassI	290,732	88,567,402	7.84
	ClassI/DIRS	900	59,454	0.01
	ClassI/LARD	37,500	10,744,331	0.95
	ClassI/LINE	198,103	62,215,959	5.51
	ClassI/LTR/Copia	425	341,007	0.03
	ClassI/LTR/Gypsy	5,781	494,569	0.04
	ClassI/LTR/Unknown	40,025	17,693,529	1.57
	ClassI/PLE	5,619	3,729,504	0.33
	ClassI/SINE	1,536	215,284	0.02
	ClassI/TRIM	176	145,812	0.01
	ClassI/Unknown	667	372,285	0.03
	ClassII	37,383	2,451,902	0.22
	ClassII/Crypton	599	36,739	0
	ClassII/Helitron	2,116	149,572	0.01
	ClassII/MITE	19	12,097	0
	ClassII/Maverick	1,278	127,223	0.01
	ClassII/TIR	20,776	1,513,866	0.13
	ClassII/Unknown	12,595	897,072	0.08
	PotentialHostGene	296	152,756	0.01

SSR	3,051	1,648,239	0.15
Unknown	4,503	1,788,457	0.16
Total	335,965	93,487,128	8.28

Table S9. Prediction of protein-coding genes.

Species	Method	Software	Homologous species	Gene number
C. olor	Ab initio	Genscan	-	39,788
		Augustus	-	20,063
		GlimmerHMM	-	106,607
		GeneID	-	25,868
		SNAP	-	57,634
	Homology-based	GeMoMa	*Anas platyrhynchos*	15,652
			Meleagris gallopavo	18,204
			Gallus gallus	16,103
			Anser cygnoides	15,077
	**Integration**	**EVM**	**-**	**17,759**
C. atratus	Ab initio	Genscan	-	37,478
		Augustus	-	20,220
		GlimmerHMM	-	120,741
		GeneID	-	25,744
		SNAP	-	60,844
	Homology-based	GeMoMa	*Anas platyrhynchos*	15,689
			Meleagris gallopavo	18,208
			Gallus gallus	16,156
			Anser cygnoides	15,097
	**Integration**	**EVM**	**-**	**17,835**

Table S10. Information of protein coding prediction genes.

	**Cygnus olor**	**Cygnus atratus**
GeneNum	17,759	17,835
Genelen	339,431,319	338,737,679
AveGenlen	19,113.20	18,992.86
ExonLen	29,120,988	29,120,034
AveExonLen	1,639.79	1,632.75
ExonNum	175,576	175,327
AveExonNum	9.89	9.83
CDSLen	29,120,988	29,120,034
AveCDSlen	1,639.79	1,632.75
CDSNum	175,576	175,327
AveCDSNum	9.89	9.83
IntronLen	310,310,331	309,617,645
AveIntronLen	17,473.41	17,360.11
IntronNum	157,817	157,492
AveIntronnum	8.89	8.83

Table S11. Prediction results of noncoding RNA.

Species	RNA classification	Number	Family
C. olor	miRNA	207	97
	rRNA	128	4
	tRNA	376	23
C. atratus	miRNA	215	101
	rRNA	130	4
	tRNA	369	23

Table S12. Functional annotation of protein-coding genes.

Species	Database	Annotated number	Percentage (%)
C. olor	GO	7431	41.84
	KEGG	11116	62.59
	KOG	11916	67.1
	TrEMBL	16900	95.16
	NR	16975	95.59
	All annotated	16988	95.66
C. atratus	GO	7409	41.54
	KEGG	11143	62.48
	KOG	11939	66.94
	TrEMBL	16963	95.11
	NR	17027	95.47
	All annotated	17048	95.59

Table S13. Summary of the gene family analysis results for 11 species.

Species	Genes number	Genes in families	Unclustered genes	Family number	Unique families	Unique genes	Average genes per family
A.platyrhynchos	16,765	16,028	737	12,572	62	190	1.27
C.moschata	15,898	15,253	645	12,897	4	9	1.18
O.jamaicensis	16,417	15,786	631	13,025	40	83	1.21
A.fuligula	15,565	15,403	162	12,679	16	51	1.21
A.cygnoides	15,439	14,956	483	12,662	3	6	1.18
A.brachyrhynchus	15,544	15,044	500	12,748	1	2	1.18
A.semipalmata	15,749	15,260	489	13,063	11	22	1.17
C.torquata	15,083	14,459	624	12,557	7	14	1.15
G.gallus	16,779	16,003	776	12,602	70	302	1.27
C.olor	17,759	16,649	1,110	14,052	3	7	1.18
C.atratus	17,835	16,694	1,141	14,037	22	51	1.19

Table S14. The statistics information of SVs in *C.olor* and *C.atratus*.

Type of SVS	Size range	Count	Total bp
Insertion	50-500 bp	4873	752195
	500-10,000 bp	3444	5172785
	Total	8317	5924980
Deletion	50-500 bp	5232	784994
	500-10,000 bp	2963	5054219
	Total	8195	5839213
Tandem expansion	50-500 bp	372	89800
	500-10,000 bp	323	857255
	Total	695	947055
Tandem contraction	50-500 bp	506	116648
	500-10,000 bp	329	762915
	Total	835	879563
Repeat expansion	50-500	796	173850
	500-10,000	913	2232519
	Total	1709	2406369
Repeat contraction	50-500	876	174317
	500-10,000	757	1809839
	Total	1633	1984156
Total		21384	

Table S15. The information of positive selected genes.

GeneID	2Δ*lnL*	P value	Gene name
Cygnus olor			
EVM0000599.1	8.069412	4.50E-03	ATAD5
EVM0001180.1	9.492328	2.06E-03	HEATR6
EVM0001472.1	7.640908	5.71E-03	ZNF804A
EVM0003170.1	7.58529	5.88E-03	GALNS
EVM0003193.1	6.749794	9.38E-03	BRCA1
EVM0003231.1	11.27513	7.86E-04	B4GALNT3
EVM0003756.1	8.10541	4.41E-03	HPDL
EVM0003861.1	6.28702	1.22E-02	FAHD2A
EVM0005510.1	7.2756	6.99E-03	NA
EVM0006686.1	18.457366	1.74E-05	SNAPC3
EVM0007654.1	14.914106	1.13E-04	FNDC1
EVM0008183.1	7.452896	6.33E-03	FAM172A
EVM0008228.1	6.580366	1.03E-02	LEO1
EVM0009007.1	8.894462	2.86E-03	RASD1
EVM0011232.1	6.766208	9.29E-03	NA
EVM0011487.1	9.431608	2.13E-03	IQCA1
EVM0013181.1	6.685916	9.72E-03	DCDC2
EVM0013323.1	7.401326	6.52E-03	LDAH
EVM0013568.1	7.351338	6.70E-03	SH3KBP1
EVM0014344.1	9.863006	1.69E-03	NAXE
EVM0014912.1	8.50467	3.54E-03	CEP164
EVM0015163.1	7.233686	7.15E-03	DYX1C1
EVM0015735.1	14.769438	1.21E-04	PDLIM7
EVM0015737.1	8.859608	2.92E-03	TDO2
EVM0016544.1	5.874436	1.54E-02	GRAMD2B
EVM0017354.1	5.80324	1.60E-02	ZNF516
Cygnus atratus			
EVM0000527.1	7.856818	5.06E-03	PNPT1
EVM0001431.1	5.098776	2.39E-02	GALNT15
EVM0001536.1	7.692264	5.55E-03	TTC19
EVM0001881.1	6.557388	1.04E-02	STK31
EVM0001970.1	6.763134	9.31E-03	NA
EVM0002810.1	4.719872	2.98E-02	ZNF516
EVM0002978.1	8.939984	2.79E-03	ZNF335
EVM0003067.1	4.438716	3.51E-02	IQCH
EVM0003488.1	7.087948	7.76E-03	CENPC
EVM0003604.1	5.368108	2.05E-02	DCBLD1
EVM0004383.1	7.63194	5.73E-03	SERINC2
EVM0006379.1	5.660372	1.74E-02	NOL8
EVM0008448.1	8.931096	2.80E-03	C26H6orf89
EVM0009914.1	5.74314	1.66E-02	ISG20

EVM0010230.1	5.66613	1.73E-02	GPRIN2
EVM0011043.1	3.880702	4.88E-02	OLAH
EVM0011989.1	6.390094	1.15E-02	CSTF3
EVM0012156.1	4.217618	4.00E-02	GTF2IRD1
EVM0012722.1	10.021572	1.55E-03	VWA9
EVM0013170.1	6.944142	8.41E-03	PLEKHG7
EVM0015731.1	12.795864	3.47E-04	ELP1
EVM0016054.1	9.306762	2.28E-03	BFSP2
EVM0017167.1	7.63352	5.73E-03	FOS
EVM0017335.1	9.329928	2.25E-03	TTLL9
EVM0017511.1	3.913726	4.79E-02	REXO5
EVM0017814.1	5.703248	1.69E-02	ZNF469
Cygnus olor + Cygnus atratus			
EVM0000333.1	5.085622	2.41E-02	CCDC170
EVM0000469.1	8.631904	3.30E-03	Sla2
EVM0000793.1	8.017968	4.63E-03	NBR1
EVM0000976.1	10.590808	1.14E-03	AGA
EVM0001137.1	6.803354	9.10E-03	BLOC1S2
EVM0002221.1	4.314614	3.78E-02	CAV2
EVM0002636.1	6.83858	8.92E-03	POLH
EVM0003015.1	6.104464	1.35E-02	KAZN
EVM0003909.1	6.217668	1.26E-02	MKRN2
EVM0004076.1	4.951694	2.61E-02	KIAA1524
EVM0004316.1	7.900574	4.94E-03	FANCC
EVM0004329.1	7.633502	5.73E-03	EXOC8
EVM0004437.1	10.733814	1.05E-03	PPID
EVM0005461.1	7.88307	4.99E-03	TMEM200B
EVM0006221.1	8.974244	2.74E-03	SYNDIG1
EVM0006411.1	6.603334	1.02E-02	TASOR
EVM0006909.1	5.31025	2.12E-02	PSPC1
EVM0007014.1	5.703776	1.69E-02	LRRC6
EVM0007571.1	3.933588	4.73E-02	PTHLH
EVM0008551.1	5.27517	2.16E-02	GPC3
EVM0008737.1	5.568092	1.83E-02	IQCG
EVM0009121.1	9.487864	2.07E-03	CD36
EVM0010775.1	4.249474	3.93E-02	ASPM
EVM0011058.1	8.181358	4.23E-03	ATXN10
EVM0011748.1	3.842534	5.00E-02	MELTF
EVM0011940.1	5.8682	1.54E-02	OLFM4
EVM0011983.1	6.176676	1.29E-02	PQLC2
EVM0012310.1	7.173148	7.40E-03	FBXL5
EVM0012655.1	9.180586	2.45E-03	PLEKHG7
EVM0012977.1	7.651182	5.67E-03	FAS
EVM0013075.1	8.359562	3.84E-03	NA
EVM0013127.1	3.891078	4.85E-02	SCNN1B
EVM0013370.1	10.204356	1.40E-03	EVX1

EVM0013884.1	9.31654	2.27E-03	RASL10B
EVM0014068.1	5.662906	1.73E-02	COG8
EVM0014738.1	6.813498	9.05E-03	CCDC18
EVM0014952.1	7.16969	7.41E-03	PTER
EVM0016309.1	4.89757	2.69E-02	AATF
EVM0016634.1	5.376568	2.04E-02	PBRM1
EVM0017347.1	18.469044	1.73E-05	MCM2

Table S16. The results of GO enrichment analyses (g:Profiler) for the positive selected genes of *C.olor* and *C.atratus*.

Groups	Functional category	Term description	Term Type	FDR	No.of genes involved
**Cygnus olor**	GO:0016701	oxidoreductase activity, acting on single donors with incorporation of molecular oxygen	molecular_function	3.70E-03	2
	GO:0016702	oxidoreductase activity, acting on single donors with incorporation of molecular oxygen, incorporation of two atoms of oxygen	molecular_function	3.70E-03	2
	GO:0003674	molecular_function	molecular_function	2.17E-02	20
	GO:0051213	dioxygenase activity	molecular_function	2.17E-02	2
**Cygnus atratus**	GO:0000175	3'-5'-exoribonuclease activity	molecular_function	7.92E-03	2
	GO:0016796	exonuclease activity, active with either ribo- or deoxyribonucleic acids and producing 5'-phosphomonoesters	molecular_function	7.92E-03	2
	GO:0004532	exoribonuclease activity	molecular_function	7.92E-03	2
	GO:0016896	exoribonuclease activity, producing 5'-phosphomonoesters	molecular_function	7.92E-03	2
	GO:0008408	3'-5' exonuclease activity	molecular_function	7.92E-03	2
	GO:1990837	sequence-specific double-stranded DNA binding	molecular_function	7.92E-03	5
	GO:0003676	nucleic acid binding	molecular_function	7.92E-03	9
	GO:0097159	organic cyclic compound binding	molecular_function	7.92E-03	11
	GO:1901363	heterocyclic compound binding	molecular_function	7.92E-03	11
	GO:0003690	double-stranded DNA binding	molecular_function	7.99E-03	5
	GO:0004527	exonuclease activity	molecular_function	8.64E-03	2
	GO:0043565	sequence-specific DNA binding	molecular_function	8.64E-03	5
	GO:0000987	cis-regulatory region sequence-specific DNA binding	molecular_function	9.11E-03	4
	GO:0000976	transcription cis-regulatory region binding	molecular_function	1.79E-02	4
	GO:0001067	transcription regulatory region nucleic acid binding	molecular_function	1.79E-02	4
	GO:0003677	DNA binding	molecular_function	3.53E-02	5
	GO:0000978	RNA polymerase II cis-regulatory region sequence-specific DNA binding	molecular_function	3.98E-02	3
	GO:0004540	ribonuclease activity	molecular_function	4.01E-02	2

	GO:0000977	RNA polymerase II transcription regulatory region sequence-specific DNA binding	molecular_function	4.93E-02	3
**Cygnus olor + Cygnus atratus**	GO:0048856	anatomical structure development	biological_process	1.38E-04	18
	GO:0032502	developmental process	biological_process	1.38E-04	19
	GO:0009987	cellular process	biological_process	1.38E-04	34
	GO:0071840	cellular component organization or biogenesis	biological_process	7.00E-04	19
	GO:0048869	cellular developmental process	biological_process	7.31E-04	14
	GO:0030154	cell differentiation	biological_process	7.31E-04	14
	GO:0008150	biological_process	biological_process	7.31E-04	34
	GO:0016043	cellular component organization	biological_process	1.02E-03	18
	GO:0065007	biological regulation	biological_process	1.02E-03	26
	GO:0005515	protein binding	molecular_function	3.44E-03	22
	GO:0005488	binding	molecular_function	3.44E-03	28
	GO:0006801	superoxide metabolic process	biological_process	3.62E-03	3
	GO:0003674	molecular_function	molecular_function	5.61E-03	32
	GO:0005516	calmodulin binding	molecular_function	7.16E-03	3
	GO:0048468	cell development	biological_process	9.23E-03	9
	GO:0019430	removal of superoxide radicals	biological_process	1.10E-02	2
	GO:0090322	regulation of superoxide metabolic process	biological_process	1.10E-02	2
	GO:0050896	response to stimulus	biological_process	1.10E-02	19
	GO:0071450	cellular response to oxygen radical	biological_process	1.11E-02	2
	GO:0071451	cellular response to superoxide	biological_process	1.11E-02	2
	GO:0044085	cellular component biogenesis	biological_process	1.11E-02	11
	GO:0050794	regulation of cellular process	biological_process	1.16E-02	22
	GO:0000305	response to oxygen radical	biological_process	1.22E-02	2
	GO:0000303	response to superoxide	biological_process	1.22E-02	2
	GO:0071214	cellular response to abiotic stimulus	biological_process	1.22E-02	4
	GO:0104004	cellular response to environmental stimulus	biological_process	1.22E-02	4

GO:0006996	organelle organization	biological_process	1.22E-02	12
GO:0051716	cellular response to stimulus	biological_process	1.22E-02	17
GO:0048523	negative regulation of cellular process	biological_process	1.30E-02	13
GO:0022617	extracellular matrix disassembly	biological_process	1.38E-02	2
GO:0030282	bone mineralization	biological_process	1.41E-02	3
GO:0048518	positive regulation of biological process	biological_process	1.47E-02	15
GO:0097150	neuronal stem cell population maintenance	biological_process	1.68E-02	2
GO:0007017	microtubule-based process	biological_process	1.68E-02	6
GO:0022607	cellular component assembly	biological_process	1.68E-02	10
GO:0048522	positive regulation of cellular process	biological_process	1.75E-02	14
GO:0032501	multicellular organismal process	biological_process	1.75E-02	15
GO:0031214	biomineral tissue development	biological_process	1.76E-02	3
GO:0050789	regulation of biological process	biological_process	1.83E-02	22
GO:0000226	microtubule cytoskeleton organization	biological_process	1.84E-02	5
GO:0110148	biomineralization	biological_process	1.89E-02	3
GO:0051179	localization	biological_process	2.28E-02	15
GO:0010646	regulation of cell communication	biological_process	2.51E-02	10
GO:0023051	regulation of signaling	biological_process	2.54E-02	10
GO:0060284	regulation of cell development	biological_process	3.19E-02	4
GO:0048519	negative regulation of biological process	biological_process	3.20E-02	13
GO:0072593	reactive oxygen species metabolic process	biological_process	3.24E-02	3
GO:0098869	cellular oxidant detoxification	biological_process	3.25E-02	2
GO:0006810	transport	biological_process	3.25E-02	12
GO:0009948	anterior/posterior axis specification	biological_process	3.43E-02	2
GO:0044458	motile cilium assembly	biological_process	3.43E-02	2
GO:0007281	germ cell development	biological_process	3.43E-02	3
GO:0010810	regulation of cell-substrate adhesion	biological_process	3.43E-02	3

GO:0045732	positive regulation of protein catabolic process	biological_process	3.43E-02	3
GO:0070925	organelle assembly	biological_process	3.43E-02	5
GO:0050793	regulation of developmental process	biological_process	3.43E-02	8
GO:0009966	regulation of signal transduction	biological_process	3.43E-02	9
GO:0051234	establishment of localization	biological_process	3.43E-02	12
GO:1990748	cellular detoxification	biological_process	3.68E-02	2
GO:0007275	multicellular organism development	biological_process	4.11E-02	11
GO:0051247	positive regulation of protein metabolic process	biological_process	4.16E-02	6
GO:0097237	cellular response to toxic substance	biological_process	4.22E-02	2
GO:1900024	regulation of substrate adhesion-dependent cell spreading	biological_process	4.22E-02	2
GO:0098754	detoxification	biological_process	4.49E-02	2
GO:0048513	animal organ development	biological_process	4.49E-02	9
GO:0045595	regulation of cell differentiation	biological_process	4.59E-02	6
GO:0048583	regulation of response to stimulus	biological_process	4.59E-02	10
GO:0035082	axoneme assembly	biological_process	4.71E-02	2
GO:0045807	positive regulation of endocytosis	biological_process	4.71E-02	2
GO:0051702	biological process involved in interaction with symbiont	biological_process	4.71E-02	2
GO:0062197	cellular response to chemical stress	biological_process	4.71E-02	3
GO:0045596	negative regulation of cell differentiation	biological_process	4.71E-02	4
GO:0009653	anatomical structure morphogenesis	biological_process	4.71E-02	8
GO:0045229	external encapsulating structure organization	biological_process	4.84E-02	3
GO:0030198	extracellular matrix organization	biological_process	4.84E-02	3
GO:0043408	regulation of MAPK cascade	biological_process	4.84E-02	4
GO:0043062	extracellular structure organization	biological_process	4.91E-02	3
GO:0009798	axis specification	biological_process	4.95E-02	2
GO:0030317	flagellated sperm motility	biological_process	4.95E-02	2
GO:0010769	regulation of cell morphogenesis involved in differentiation	biological_process	4.95E-02	2

GO:0034644	cellular response to UV	biological_process	4.95E-02	2
GO:0097722	sperm motility	biological_process	4.95E-02	2
GO:0022412	cellular process involved in reproduction in multicellular organism	biological_process	4.95E-02	3
GO:0045862	positive regulation of proteolysis	biological_process	4.95E-02	3
GO:0003006	developmental process involved in reproduction	biological_process	4.95E-02	4
GO:0080134	regulation of response to stress	biological_process	4.95E-02	5
GO:0010647	positive regulation of cell communication	biological_process	4.95E-02	6
GO:0023056	positive regulation of signaling	biological_process	4.95E-02	6
GO:0009605	response to external stimulus	biological_process	4.95E-02	7
GO:0051128	regulation of cellular component organization	biological_process	4.95E-02	7
GO:0002376	immune system process	biological_process	4.95E-02	7
GO:0006950	response to stress	biological_process	4.95E-02	9
GO:0023052	signaling	biological_process	4.95E-02	13

Table S17. Oligonucleotides Used for WISH Probe.

Gene	Forward primer 5′-3′	Reverse primer 5′-3′	Length
PWWP2A	CCGGTCACTCATCTCATCGG	GAGCGTGGATCACTACGAGG	834

Table S18. sgRNA Core Sequence and Nested Primers.

Gene	Primer	Sequence 5′-3′
PWWP2A	sgRNA1	TAATACGACTCACTATAGGAAAATGGCGGCCGTGGCT GGTTTTAGAGCTAGAAATAGC
	sgRNA2	TAATACGACTCACTATAGGTGCGGAGCCAGGAGCTGC TGGTTTTAGAGCTAGAAATAGC
	Forward primer	GCCAAAGTTGCATCCTCATAA
	Reverse primer	GGCCTTCTGATTTCCATTTG

Table S19. RT-PCR primers.

Gene	Forward primer 5′-3′	Reverse primer 5′-3′	Length
PWWP2A	ACCTCTCGAGAAGGTTCGGAC	TGTCTCTGACTGGAATGCCTCCAT	116
TYR	AACGCGCTGGAAGGTTTTGC	TTGGCAGATCCCTGCACTGAAGA	122
TYRP1a	AGCGCGACATGCAGGATATG	TTGGAGCTGATGGCAGTGGAGT	145
TYRP1b	AGCGCGACATGCAGGATATG	TTGGAGCTGATGGCAGTGGAGT	145
DCT	AGACACGCTTCTGGGTCCTG	AAGTTCTCATTACCCGTCAGTTTCTG	138
